# The Southern Presbyterian

**Published:** 1878-11-20

**Authors:** 


THE SOUTHERN PRESBYTERIAN.—Edited by J. W. Woodrow D. D. and published in Columbia, S. C., at $3.00 per annum, is one of the best religious newspapers on the continent. It is conducted with much ability, and contains both general and religious information.

